# Prevalence and determinants of female genital amputation among adolescent girls and young women in Ethiopia: multilevel analysis

**DOI:** 10.1186/s41043-023-00484-1

**Published:** 2023-12-15

**Authors:** Asaye Alamneh Gebeyehu, Rahel Mulatie Anteneh, Anteneh Mengist Dessie, Chalachew Yenew

**Affiliations:** Department of Public Health, College of Health Sciences, Debre Tabor University, Debre Tabor, Ethiopia

**Keywords:** Adolescent, Female genital amputation, Women, Ethiopia

## Abstract

**Introduction:**

Female genital amputation is a harmful traditional practice that has adverse risks on health outcomes. Consequently, it affects girls and women's physical, psychological, and mental health conditions. However, evidence on female genital amputation factors among adolescent girls and young women in Ethiopia was limited.

**Method:**

Secondary data analysis was conducted from the 2016 Ethiopian Demographic and Health Survey. A weighted sample size included in this study was 2961 adolescent girls and young women. Data management and further analysis were performed using Stata 14 software. An adjusted odds ratio with a 95% confidence interval was used for measuring a significant relationship between factors with the outcome variable.

**Result:**

This study found that the prevalence of female genital amputation among adolescent girls and young women in Ethiopia was 53.4%. Among individual- and community-level variables included in the multivariable multilevel analysis: maternal age, religious status, marital status, maternal educational level, occupational status, residence areas, community uneducated level, and community mass media were significant factors for female genital amputation.

**Conclusion:**

The prevalence of female genital amputation among adolescent girls and young women in Ethiopia remains high. Those individual- and community-level factors influence female genital amputation among adolescent girls and young women in Ethiopia. It requires health interventions on female genital amputation to improve behavioral changes and create awareness about harmful practices.

## Introduction

Female genital amputation (FGA) is the partial or complete removal of the female external genitalia or injuring genital organs without any non-medical reasons [1]. FGA practices violate women's right to life and have serious negative consequences for their physical, mental, and social health outcomes [2]. It also has short- and long-term adverse effects on maternal and childbirth health outcomes [3, 4]. FGA practices are highly related to short-term health risks such as bleeding, shock, severe pain, difficulty controlling urine, and infection. In the long-term effect, girls and women who have undergone FGA are also at risk of suffering from childbirth complications throughout their life, chronic pelvic pain, urinary tract infections, and post-traumatic stress disorder (PTSD) [5, 6]. Globally, more than 200 million girls and women have undergone FGA practice in 30 countries, including Africa, the Middle East, and Asia; more than 3 million girls are at risk for FGA annually [7]. In Ethiopia, 25 million girls and women have experienced FGM [8]. Not only infants but also adolescent girls and young women are circumcised.

FGA is a common and harmful practice in Ethiopian communities. There are various reasons to practice FGA procedures, which vary across communities. FGA practice is not being phased out due to cultural and religious norms [9, 10]. In many societies, FGM is perceived to protect premarital sexual activity, reduce early pregnancy, control women's emotions, and preserve chastity and cultural requirements [11–13]. Each country has laws and policies to combat FGM, including legislation to protect girls' right to health and gender-based discrimination and violence, including FGM [14]. In the Federal Democratic Republic of Ethiopia, laws and policies have been raised to protect women's rights in the home and prevent harmful practices like female genital mutilation (FGM), child marriage, and other types of gender-based violence [15]. In 2013, the Ethiopian government implemented national strategies and actions regarding the harmful traditional practices against adolescent girls and women and had a great chance to significantly create awareness about the health risks of FGM, change community-led mobilization, improve access to essential health services, and protect against sexual violations [16–18]. Additionally, community-based education interventions can help women to understand the adverse health risks of practicing FGA [19]. The Ethiopian government has set a goal to eliminate FGA by 2025 [20], but despite legal and policy restrictions, FGA is still being practiced.

Despite national efforts, the prevalence of FGA remains high in Ethiopia [21, 22]. Previous studies in Ethiopia have focused on the prevalence and individual-level factors of female genital amputation in specific areas [23–27]. However, this study uses national and large-scale datasets to identify individual- and community-level variables of female genital amputation among adolescent girls and young women. Therefore, the study aimed to determine the prevalence and identify factors associated with FGA practices among adolescent girls and young women in Ethiopia using multilevel analysis.

## Methods

### Study settings

The dataset on women was obtained from the 2016 Ethiopia Demographic and Health Survey (EDHS), and this was the fourth national representativeness survey. Ethiopia is one of the eastern Horn African countries and is located between 3° and 15° north latitude and 33° and 48° east longitude [18]. The country has comprised of nine regional states and two administrative cities. Each administrative state is subdivided into various zones; each zone is divided into different districts (woreda), and the woreda is divided into sub-portion administrative units called kebele. According to the 2007 Ethiopian Population and Housing Census (PHC), use two-stage stratified sampling to obtain nationally representative relevant information for key health indicators. An average of 181 households is covered per enumeration area (EAs) in a complete enumeration. Each regional state was stratified independently and separately into urban and rural areas. The sample selected from the enumeration area (EAs) was independent, with probability proportional to EAs size in each stratum.

According to the 2007 PHC, the sample was selected using the two stages. In the first stage, 645 EAs from all 84,915 listed enumeration areas (202 EAs in urban areas and 443 EAS in urban areas) were selected independently with proportional allocation to EAs size. In the second stage, the complete listing of households in each selected cluster was used as a sampling frame; on average, 28 households per cluster were randomly selected with an equal probability. From all eligible households, 15,683 reproductive-aged women completed the interviews with a 95% response rate. The detailed sampling methodology has been described in the 2016 EDHS report [6].

### Data source, study population, and period

The relevant variables used for this study were extracted from women's files. The dataset was obtained from the 2016 Ethiopian Demographic and Health Survey (EDHS), and the survey data was collected from January 18 to June 27, 2016. All adolescent girls and young women aged 15–24 years from all selected clusters in Ethiopia were considered the study population.

### Study variables

#### Dependent variable

The outcome or dependent variable in this study was experiencing female genital amputation; it encompasses three parameters whether the respondents experienced FGA or supported continuation of FGA or whether their daughters had undergone a genital amputation. The response variable was coded as "1" if adolescent girls and young women had been circumcised and "0" if they had not been circumcised.

#### Independent variables

For this study, we classify the independent variables of female genital amputation into individual- and community-level variables. Individual-level variables include demographic characteristics, whereas community-level variables are shared characteristics among study participants about clusters. In this study, community-level variables have been generated by aggregating individual-level variables such as media exposure, maternal education, and wealth index. If the assumption of normal distribution fails, the median is preferable to classify the aggregated community-level variables into "high" and "low" groups regarding the median values of the proportion.

### Data management and statistical analysis

Data cleaning, extracting, and recoding were performed using STATA version 14. Sampling weights were used to adjust the non-response rate and sampling designs to restore the national representativeness sample to get valid statistical estimates.

In this study, both summary and inferential statistics were performed to analyze the data. We made summary statistics to compute frequency and percentage background characteristics. Multilevel logistic analysis was used to identify significant determinants of female genital amputation by accounting for the hierarchical data nature. Adolescent girls and young women aged 15–24 were nested within clusters, and the clusters were used as random effects to account for unexplained variability at the community level. After selecting relevant variables for this study, multilevel logistic analysis was employed to obtain valid statistical estimates by accounting for the cluster effects. Before conducting the final model, a bivariable multilevel logistic regression analysis was done to determine a relationship between the outcome variable and an independent variable. Those explanatory variables with a p-value less than 0.25 were considered in multivariable multilevel analysis. Four possible fitted models were performed with hierarchical data: the null model with no explanatory variables, the second model with only individual-level variables, the third model with only community-level variables, and the final (fourth) model consist of both individual- and community-level variables. The better-fitted model with the data from these four models was selected using a lower deviance value. Adjusted odds ratio (AOR) with a 95% confidence interval was employed to measure a significant relationship between independent variables with female genital amputation. Intra-class correlation (ICC), a proportional change in variance (PCV), and median odds ratio (MOR) were statistical parameters to measure cluster heterogeneity or random effects. Intra-class correlation (ICC) is used to measure the similarity of individual values within a cluster, and it is also computed as $\mathrm{ICC}=\frac{{{\updelta }^{2}}_{\mathrm{U}0}}{{{\updelta }^{2}}_{\mathrm{U}0}+{{\updelta }^{2}}_{\upvarepsilon }}$ where ${{\delta }^{2}}_{\varepsilon }=\frac{{\pi }^{2}}{3}=3.29$ is the residual variance for individual-level units that have a logistic distribution and ${{\delta }^{2}}_{U0}$ is the variation between clusters in the null model.

Proportional change in variance (PCV) is also used to determine the extent of explained variability in the model by adding possible explanatory variables, and its formula would be $\mathrm{PCV}=\frac{{\upsigma }_{\mathrm{U}}^{2}\left[{\mathrm{Model}}_{\mathrm{Null}}-{\mathrm{Model}}_{\mathrm{full}}\right]}{{\upsigma }_{\mathrm{U}}^{2}[{\mathrm{Model}}_{\mathrm{Null}}]}*100$; where ${\upsigma }_{\mathrm{U}}^{2}$ represents the variance of the model, ${\mathrm{Model}}_{\mathrm{Null}}$ is a null model, and ${\mathrm{Model}}_{\mathrm{Full}}$ is a full model [28].

MOR indicates the odds ratio of an individual from a higher-risk group with female circumcision in one cluster and a lower-risk group with female circumcision in the other cluster when two individuals were randomly picked from two groups. The MOR value can be calculated as $\mathrm{MOR}=\mathrm{exp}\left(\sqrt{2*{\mathrm{V}}_{\mathrm{A}}}*0.6745\right)\approx \mathrm{exp}\left(0.95\sqrt{{\mathrm{V}}_{\mathrm{A}}}\right)$; where ${\mathrm{V}}_{\mathrm{A}}$ is the variance of a higher level, and 0.6475 is the 75th commutative distribution [29].

### Ethical consideration

The dataset in this study was accessed and downloaded from the measure DHS program by submitting the aim of the study through the website http://www.dhsprogram.com. The datasets used for this study were kept confidential, and any participant identifier responses during data collection were removed from the study. Detailed information about methodology and ethical issues was published in the final 2016 EDHS report [6].

## Results

### Background characteristics of study participants

The analysis for this study included a total sample of 2961 women between the ages of 15 and 24. The mean age of the study participants was approximately 19 years. Of all study participants, 1671 (56.4%) were adolescent girls and young women aged between 15 and 19 (Table 1). Based on the sex of the household head, 2200 (74.3%) of adolescent girls and young women were led by males. The majority of the respondents, 1307 (44.14%), were Orthodox, and 868 (29.31%) were Muslim religious followers. Regarding marital status, 1709 (57.73%) and 1081 (36.5%) of these women were single and married, respectively. Out of all participants in the study, 2263 (76.43%) adolescent girls and young women lived in rural areas. Based on the educational level of women, 551(18.6%) women had no formal education, and 1650 (55.71%) women had a primary school. According to wealth index status, 1003 (33.8%) of adolescent girls and young women were from the poor wealth index, and 1610 (54.36%) were not working. Of these respondents, 850 (67.89%) of adolescent girls and young women got married before turning 18, and 1451 (49.01%) of these women had no media exposure. Regarding regions, 1082 (36.5%) of all adolescent girls and young women were from Oromia, and 656 (22.17%) were from the Amhara region. Moreover, 1170 (39.5%) and 1539 (51.96%) of the participants had a low proportion of community poverty and an uneducated community, respectively (Table 1).

**Table 1:** Background characteristics of adolescent girls and young women in Ethiopia, 2016

Variables	Categories	Frequency	Percent (%)
Age	15–19	1671	56.4
	20–24	1290	43.6
Sex of household head	Male	2200	74.3
	Female	761	25.7
Religion	Orthodox	1307	44.14
	Muslim	868	29.31
	Protestant	710	23.98
	Catholic	30	1.02
	Traditional	33	1.12
	Others	13	0.43
Marital status	Never married	1709	57.73
	Married/living with partners	1081	36.5
	Divorced/widowed/separated	171	5.77
Residence	Urban	698	23.57
	Rural	2263	76.43
Mother’s education	No education	551	18.6
	Primary	1650	55.71
	Secondary	563	19.02
	Higher	197	6.67
Husband’s education	No education	350	32.34
	Primary	517	47.86
	Secondary	151	14.01
	Higher	63	5.79
Occupation status	Not working	1610	54.36
	Working	1351	43.64
Age at first marriage	Early (< 18)	850	67.89
	Later (> 18)	402	32.11
Wealth index	Poor	1003	33.85
	Middle	547	18.48
	Rich	1411	47.66
Mass media exposure	No	1451	49.01
	Yes	1510	50.99
Region	Tigray	233	7.86
	Afar	30	1.0
	Amhara	656	22.17
	Oromia	1082	36.55
	Somalia	89	3.01
	Benishangul-Gumuz	29	0.99
	SNNP	606	20.47
	Gambella	9	0.3
	Harari	8	0.25
	Addis Ababa	199	6.71
	Dire Dewa	20	0.69
Community poverty	Low	1170	39.5
	High	1791	60.5
Community media exposure	Low	1444	48.77
	High	1517	51.23
Community poor education	Low	1539	51.96
	High	1422	48.04

### Prevalence of female genital amputation

**Fig. 1:**
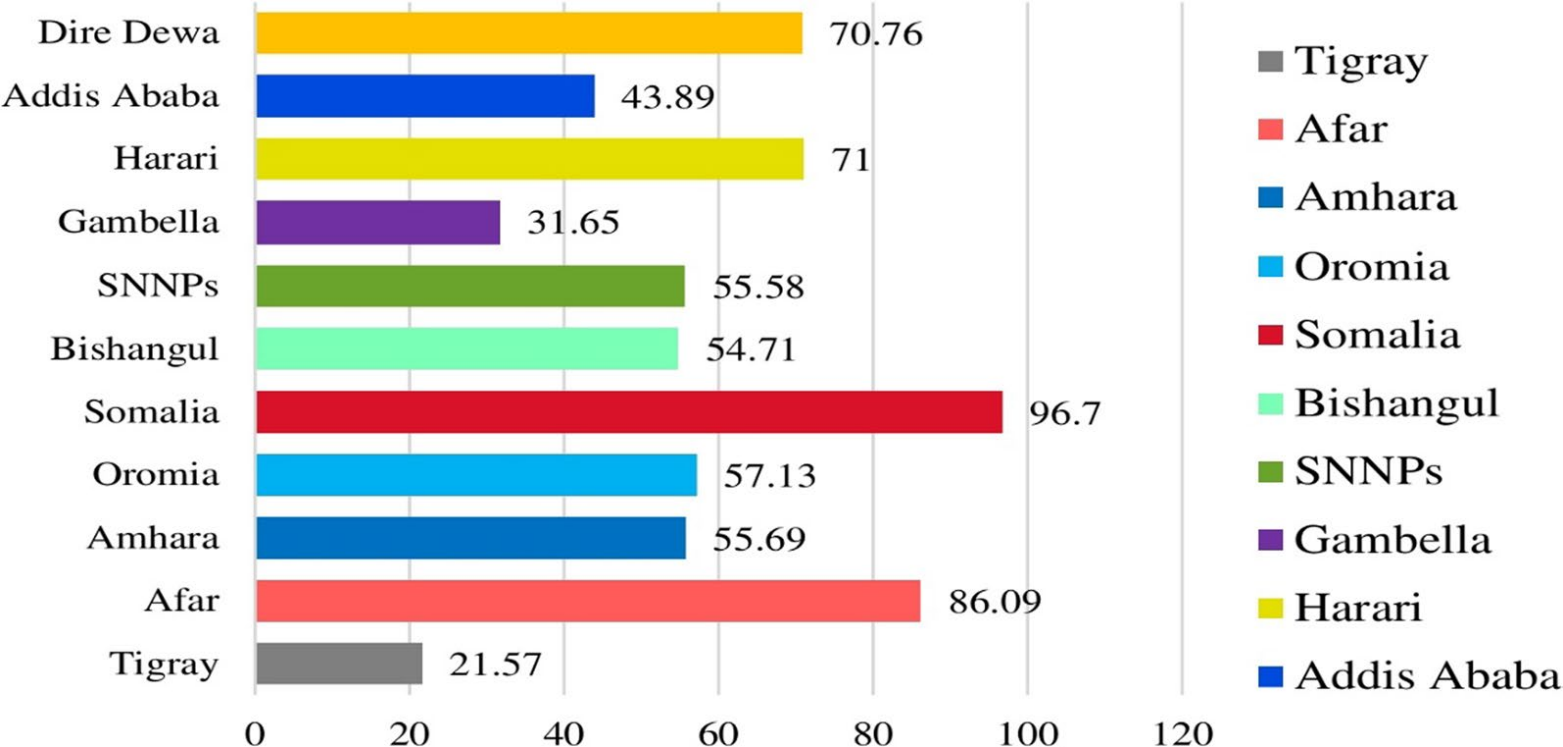
Prevalence of FGA among adolescent and young women across the region in Ethiopia, 2016

In this study, 1608(54.31%) of adolescent girls and young women in Ethiopia had their female genitalia amputated. A 95% confidence interval of prevalence lies between 52.52 and 56.11 (95% CI: 52.52, 56.11). Female genital amputation prevalence among adolescent and young women in Ethiopia varied significantly across the regions, ranging from 51(21.57%) in the Tigray region to 87(96.7%) in Somalia (Fig. 1).

### Random effects and model fits

We consider multilevel logistic analysis for accounting cluster variation because the value of ICC in model one (the null model) is higher than 5%. It reveals that 58.3% of the total variation in female genital amputation is due to community-level factors and occurs at the cluster level (Table 2). The final model (Model IV), which had the lowest deviance statistic value of the four fitted models, was a better fit with the data. The PCV valve in the best-fitting model (model IV) was 30.86%, implying that combining individual- and community-level variables explained 30.86% of the total variation. Furthermore, the median odds ratio value of 4.02 shows that a person from a cluster with a higher risk of female genital mutilation was 4.02 times more likely to practice female genital amputation than a person from another group with a lower risk of female genital amputation (Table 2).

**Table 2:** Measuring community-level variation for female genital amputation among adolescent girls and young women in Ethiopia, 2016

Random effects	Model I	Model II	Model III	Model IV
Community-level variance	4.60	2.43	3.50	2.42
ICC	58.3	42.48	51.54	42.38
PCV (%)	Reference	47.17	44.03	30.86
MOR	5.54	4.03	4.83	4.02
Log-likelihood	− 1784.31	− 1620.64	− 1727.93	− 1607.28
Deviance	3568.62	3241.28	3455.86	3214.56

### Individual- and community-level factors with female genital amputation

A multilevel logistic analysis analyzed factors at the individual and community levels. From the bivariable multilevel logistic analysis, factors (at the individual and community levels) with a *p*-value of less than 0.25 were taken into account in the multivariable multilevel logistic analysis to identify significant variables. A multivariable multilevel logistic analysis revealed that maternal age, religious affiliation, marital status, maternal educational level, occupation, place of residence, and status within the uneducated community were significant risk factors for female genital amputation (Table 3).

**Table 3:** Multivariable multilevel logistic analysis of individual- and community-level factors of female genital amputation among adolescent girls and young women in Ethiopia, 2016

Variables and their categories	Model I	Model II	Model III	Model IV
*Age*				
15–19	1		1	
20–24	1.41 (1.1, 1.79)		1.4 (1.1, 1.8)	
*Sex of household heads*				
Male	1		1	
Female	1.02 (0.81, 1.29)		1.0 (0.80, 1.28)	
*Religion*				
Orthodox	1		1	
Muslim	9.1 (6.61, 12.5)		7.3 (5.25, 10.1)	
Protestant	0.24 (0.95, 1.9)		1.32 (0.92, 1.88)	
Catholic	1.86 (0.52, 6.62)		1.75 (0.49, 6.2)	
Traditional	0.25 (0.032, 1.95)		0.2 (0.03, 1.57)	
*Marital status*				
Never married	1		1	
Married/ living with partners	1.69 (1.28, 2.21)		1.68 (1.27, 2.2)	
Divorced/windowed/separated	1.44 (0.89, 2.35)		1.49 (0.91, 2.42)	
*Maternal educational status*				
No education	1		1	
Primary	0.83 (0.59, 1.15)		0.96 (0.68, 1.36)	
Secondary	0.51 (0.34, 0.76)		0.61 (0.40, 0.92)	
Higher	0.35 (0.21, 0.56)		0.4 (0.25, 0.66)	
*Occupational status*				
Not working	1.36 (1.09, 1.7)		1.36 (1.09, 1.7)	
Working	1		1	
*Wealth index status*				
Poor	1		1	
Middle	1.02 (0.71, 1.48)		1.01 (0.69, 1.47)	
Rich	1.03 (0.74, 1.44)		1.06 (0.71, 1.58)	
*Mass media exposure*				
No	1		1	
Yes	0.87 (0.66, 1.14)		0.61 (0.56, 0.79)	
*Residence*				
Urban		1	1	
Rural		3.59 (1.91, 6.73)	2.29 (1.28, 4.1)	
*Region*				
Central		1	1	
Peripheral		4.25 (2.68, 6.71)	1.93 (1.26, 2.96)	
Metropolitans		5.01 (2.81, 8.89)	2.24 (1.31, 3.82)	
*Community uneducated status*				
Low		1	1	
High		2.91 (1.91, 4.44)	1.73 (1.16, 2.59)	
*Community poverty*				
Low		1	1	
High		0.72 (0.46, 1.12)	0.95 (0.63, 1.44)	
*Community media exposure*				
Low		1	1	
High		0.67 (0.38, 1.48)	0.56 (0.32, 1.17)	
Constant	1.7 (1.4, 2.2)	0.59 (0.38, 0.92)	0.29 (0.15, 0.54)	0.24 (0.11, 0.52)

Female genital amputation was more likely to occur in women between the ages of 20 and 24 (AOR = 1.4; 95% CI: 4.23, 7.09) than in those between the ages of 15 and 19 (Table 3). Muslim religious followers were 7.35 (AOR = 7.35; 95% CI: 5.25, 10.1) times more likely to practice female genital amputation than orthodox followers. The odds of practicing FGA were 39% (AOR = 0.61; 95% CI: 0.40, 0.92) and 0.40% (AOR = 0.40; 95% CI: 0.25, 0.66) in women who had a secondary or higher education, which was lower than adolescent girls and young women with no formal education, respectively. Women who were married or lived with partners had 1.68 (AOR = 1.68; 95% CI: 1.27–2.2) times higher odds of experiencing female genital amputation than women who were not married. Compared to women with working jobs, women with non-working jobs had higher odds of practicing FGA (Table 3). Participants who lived in rural areas were 2.29 (AOR = 2.29; 95% CI: 1.28, 4.1) times more likely to practice female genital amputation than women from urban areas. Adolescent girls and young women from semi-peripheral and metropolitan regions are 1.93 (AOR = 1.93; 95% CI: 1.26, 2.96) and 2.24 (AOR = 2.24; 95% CI: 1.31, 3.82) times more likely to practice female genital amputation than women from large central regions, respectively. Furthermore, the odds of FGA practice among adolescent girls and young women were higher in the higher uneducated community compared to the lower uneducated community (AOR = 1.73; 95% CI: 1.16, 2.59) (Table 3).

## Discussion

In this study, the prevalence of female genital amputation among adolescent girls and young women in Ethiopia was 54.31%. This finding is higher than the studies done in Hababo Guduru, Ethiopia, and Nigeria [30, 31]. However, this result was lower than the prevalence of female genital mutilation conducted at the national level in Ethiopia [32]. The possible reason could be cultural differences toward practicing female genital amputation in the community, and they may have different awareness about the risk of female genital amputation in their daughter's entire life. Adolescent girls and young women from Somalia and Afar regions experienced a higher percentage of female genital amputation might increase the overall prevalence in Ethiopia. The possible reason might be the community in the remote areas may practice harmful female genital amputation due to a lack of access to adequate information to create awareness of female genital mutilation.

Maternal age had a statistically significant effect on female genital amputation. Adolescent girls and young women aged 20–24 were 40% more likely to be circumcised than women aged between 15 and 19. This result showed that adolescent girls and young women aged between 15 and 19 years have awareness about adverse events of female genital cutting from government and non-government supportive agencies to stop FGM and improve reproductive health. However, these young women (20–24 years) may not access information regarding the disadvantage of FGM from concerned bodies, and they are highly vulnerable to practicing harmful traditional activities. The odds of practicing FGA from Muslim religious followers were 7.3 times higher than Orthodox religious followers. This finding is comparable with other studies conducted in Ethiopia, Burkina Faso, Nigeria, Kenya, and Ghana [33–38]. Place of residence also had a positive and significant relationship with female genital mutilation. Adolescent girls and young women who lived in rural areas were more likely to experience female genital amputation than women in urban areas. This finding is consistent with the studies conducted in Ethiopia, Nigeria, and Guinea [26, 30, 35, 39]; it implies that urban resident women have opportunities to access better information and have great opportunities to get awareness from different healthcare providers about harmful practices of female genital cutting.

Education is another statistically significant factor that affects female genital amputation. The odds of experiencing female genital amputation had decreased as the educational level increased. Adolescent girls and young women with secondary and higher education decreased the probability of circumcision than women with no education by 39% and 60%, respectively. The reason could be health education is one of the key pillars and indicators to improve the communities' awareness regarding the benefits of maternal health services, and educated women could have great potential to prevent harmful traditional practices. Those educated women may access updated healthcare information from mass media to build up better knowledge and ability to implement interventions on female genital mutilation. Women's occupational status was a statistically significant variable associated with female genital amputation. Adolescent girls and young women who were not working jobs had higher odds of practicing FGM than women who were working jobs by a factor of 1.36. This study is consistent with previous studies conducted in Ethiopia. Ghana, and Nigeria [32, 35, 40]. The possible reason might be women who had no work had a low economic status and were less empowered in decision-making in the household. It implies that women may not get various opportunities to condemn harmful traditional practices from people experienced in the workplace.

Mass media exposure was another independent variable that had a negative statistical impact on female genital amputation. Adolescent girls and young women who were exposed to mass media were less likely to practice female genital amputation than those who were not exposed to mass media. This finding is consistent with the studies conducted in Sub-Saharan Africa, Ethiopia, and Mogadishu, Somalia [41–43]. Mass media plays a significant role in creating awareness about various healthcare programs and health information for the communities to improve their health services utilization and to understand the negative consequences and disadvantages of harmful practices. Adolescent girls and young women exposed to mass media could have a great understanding of healthcare information, especially about the adverse events of practicing female genital mutilation on health outcomes; this indicates that they could have less chance to practice female genital mutilation.

The likelihood of practicing female genital amputation was statistically different across the regions. Adolescent girls and young women who lived in semi-peripheral areas (Afar, Somalia, Benishangul-Gumuz, and Gambella) and metropolitans (Harari, Addis Ababa, and Dire Dewa) were 1.92 and 2.24 times higher odds of practicing FGA than these women from large central regions, respectively. This result is in line with the study done in Nigeria [44], and this might be due to cultural differences and discrepancies in the norms and stems of experiencing FGA in the community.

This study showed that the odds of practicing female genital amputation were higher within communities with a high proportion of uneducated women than a low proportion of those women within the communities. The odds of practicing female genital amputation in a community with a high level of uneducated women were increased by 73% compared with a lower proportion of those women within the community. There is a norm or culture in society to support the continuation of female genital amputation. It implies that adolescent girls and young women in communities with illiteracy may not attend school and not understand the adverse effects of practicing female genital amputation on health outcomes.

### Strength, limitation, and recommendations

The strengths of this study would be the use of appropriate representative samples from large datasets and multilevel analysis to handle hierarchical data. We applied sampling methods to adjust the non-response rate and restore the nationally representative sample. The limitation of this study is that the secondary data did not measure some relevant variables during data collection, like the communities' perception of FGA, sexual initiation, and cultural norms. Furthermore, this study considered cross-sectional data, so it is impossible to determine the causality effect. We recommend future researchers conduct a mixed model to assess psychological and cultural norms.

## Conclusion

In this study, the prevalence of female genital amputation among girls and young women is still high in Ethiopia. This study revealed higher prevalence of female genital cutting or mutilation due to the continuation of harmful traditional practices and cultural beliefs. Based on the multivariable multilevel analysis: respondents' age, religion, marital status, maternal educational level, residence areas, region, and uneducated status of the community were significant variables associated with female genital amputation among girls and young women. Therefore, the Ethiopian government and non-government organizations should focus on scale-up programs for girls' higher education, rural community, and religious leaders about the risk of female genital amputation on health outcomes.

## Data Availability

The dataset used for this study was obtained from an online publicly available website of the measure DHS program by submitting the purpose of the study. The 2016 EDHS dataset was available on the public domain DHS measure (www.measuredhs.com).

## Abbreviations

CI: Confidence interval
EDHS: Ethiopian Demographic and Health Survey
EAs: Enumeration areas
FGA: Female genital amputation
USAID: US Agency for International Development
ICC: Intra-class correlation coefficient
MOR: Median odds ratio
PCR: Proportional change in variance
PHC: Population house census
